# Supplementary material for: A simple method for analyzing competitive growth of multiple cell types in xenograft tumors
Source: PLoS One. 2026 Oct 15;21(10):e0357415. doi: 10.1371/journal.pone.0357415 (PMC13671648; doi:10.1371/journal.pone.0357415)

A

One equal mix, and two mixes with reduced representation of each tag (13 total).

Tag	1	2	3	4	5	6
Mix A	10	10	10	10	10	10
Mix B	0.1	10	10	10	10	10
Mix C	0.01	10	10	10	10	10

qPCR from each for reduced representation Tag, Venus, and all six Tags from equal mix, with two input amounts (0.1ng and 0.01ng total).

B

Mix	A	B	C
Input Tag 1 (ng)	10	0.1	0.01
Input Tags 2-6 (ng ea)	10	10	10
Total input (ng)	60	50.1	50.01
Concentration (ng/ul)	10	8.35	8.335
Tag 1 (% total)	16.67	0.1996	0.02000
pg Tag 1 (0.1ng PCR)	16.7	0.167	0.0167
pg Tag 1 (0.01ng PCR)	1.67	0.0167	0.00167

C

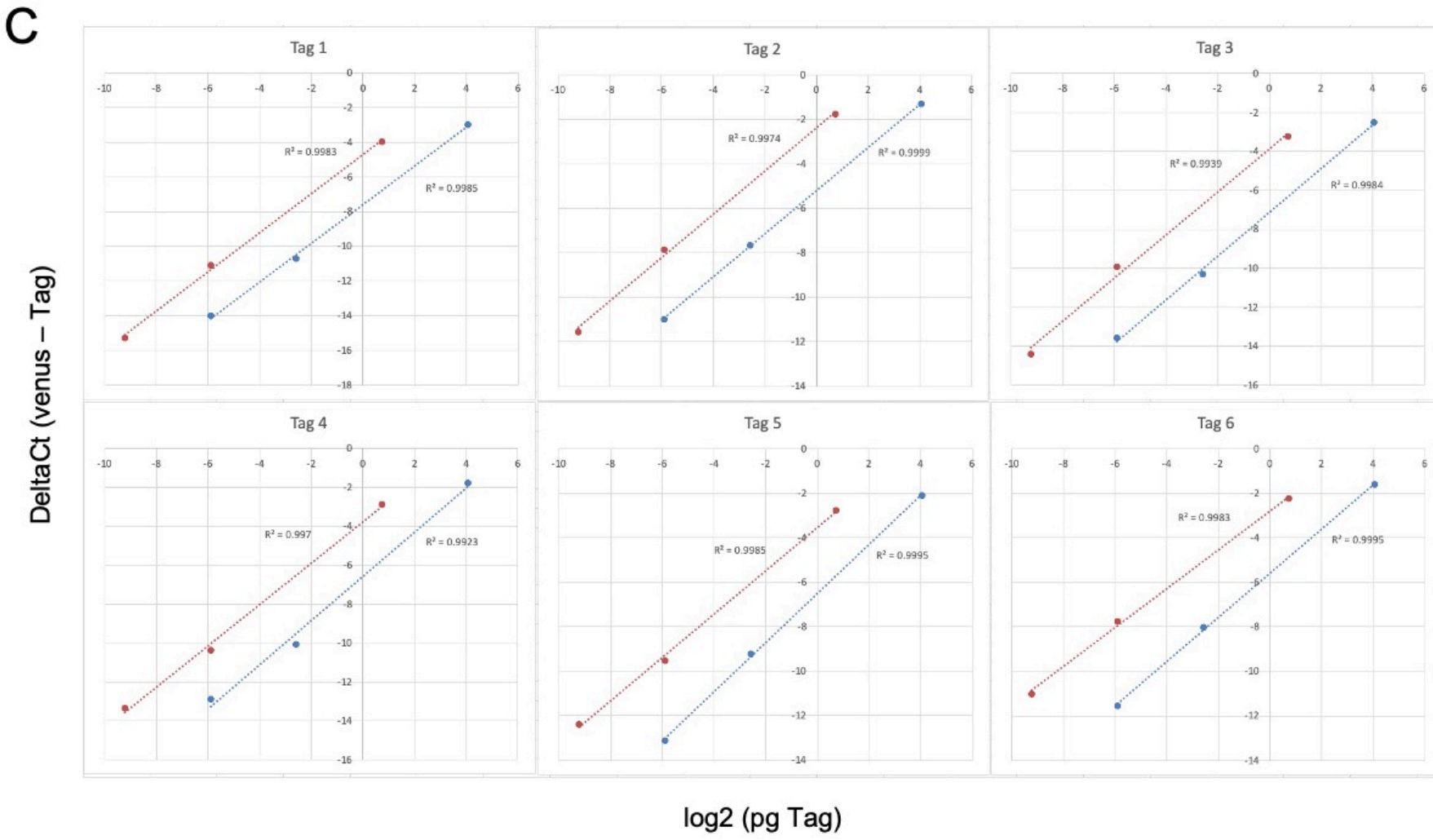

D

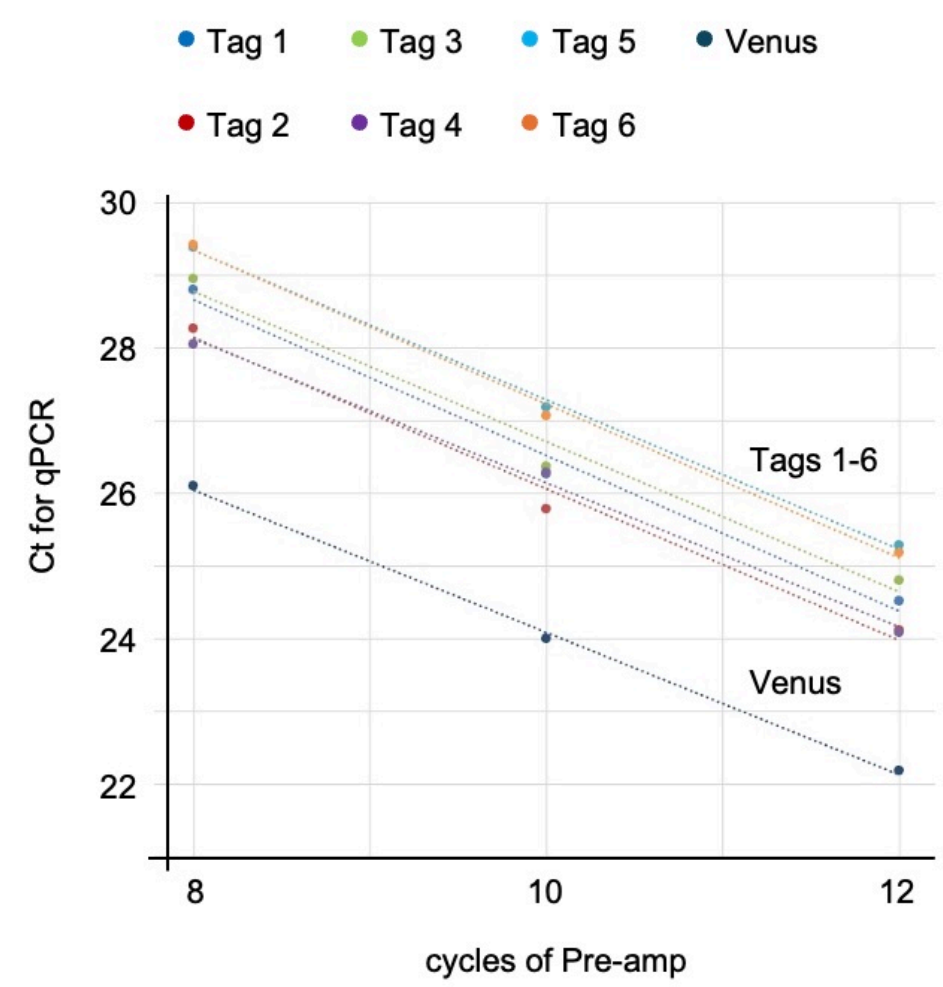

Supplement: Supplementary file 1 [file pone.0357415.s001.pdf]
