# Supplementary material for: A simple method for analyzing competitive growth of multiple cell types in xenograft tumors
Source: PLoS One. 2026 Oct 15;21(10):e0357415. doi: 10.1371/journal.pone.0357415 (PMC13671648; doi:10.1371/journal.pone.0357415)
Supplement: Supplementary file 2 [file pone.0357415.s002.pdf]

		Ct			dCt (S0-tumor)			ddCt (tag-venus)			
animal_id	tumor_id	Tag 3	Tag 6	Venus	Tag 3	Tag 6	Venus	Tag 3	Tag 6	prop T3	prop T6
1	1	30.660	30.020	28.370	-6.207	-5.183	-5.330	-0.877	0.147	0.3297	0.6703
2	2	29.220	28.690	27.220	-4.767	-3.853	-4.180	-0.587	0.327	0.3468	0.6532
2	3	29.630	29.030	27.460	-5.177	-4.193	-4.420	-0.757	0.227	0.3359	0.6641
2	4	26.590	26.110	24.730	-2.137	-1.273	-1.690	-0.447	0.417	0.3547	0.6453
2	5	24.910	24.020	23.060	-0.457	0.817	-0.020	-0.437	0.837	0.2926	0.7074
3	6	25.850	25.180	23.760	-1.397	-0.343	-0.720	-0.677	0.377	0.3252	0.6748
3	7	25.860	25.420	23.970	-1.407	-0.583	-0.930	-0.477	0.347	0.3611	0.6389
3	8	31.820	31.720	29.450	-7.367	-6.883	-6.410	-0.957	-0.473	0.4170	0.5830
4	9	26.240	26.780	24.450	-1.787	-1.943	-1.410	-0.377	-0.533	0.5271	0.4729

S0	24.453	24.837	23.040

HT29 cecal tumors
1:1 mix of Tag 3 cells (shCtrl) and Tag 6 cells (shACADM)
Four mice, with a total of 9 tumors

Mouse #	# tumors
1	1
2	4
3	3
4	1

Wilcoxson signed rank, using proportions
p-value 0.007812

ANOVA using ddCt values
p-value by animal 0.331
p-value by Tag 0.000152
