# Supplementary material for: A simple method for analyzing competitive growth of multiple cell types in xenograft tumors
Source: PLoS One. 2026 Oct 15;21(10):e0357415. doi: 10.1371/journal.pone.0357415 (PMC13671648; doi:10.1371/journal.pone.0357415)
Supplement: Supplementary file 3 [file pone.0357415.s003.pdf]

Aug 21, 2026

Detailed method for use of PCR tags

DOI
https://dx.doi.org/10.17504/protocols.io.q26g7q131lwz/v1

David Wotton[1], Tiffany Melhuish[1]
[1]University of Virginia

PCR tagged cells

David Wotton
University of Virginia

OPEN ACCESS

DOI: https://dx.doi.org/10.17504/protocols.io.q26g7q131lwz/v1

Protocol Citation:
David Wotton, Tiffany Melhuish 2026. Detailed method for use of PCR tags. **protocols.io**
https://dx.doi.org/10.17504/protocols.io.q26g7q131lwz/v1

License: This is an open access protocol distributed under the terms of the **Creative Commons Attribution License**, which permits unrestricted use, distribution, and reproduction in any medium, provided the original author and source are credited

Protocol status: Working
We use this protocol and it's working

Created: August 05, 2026

Last Modified: August 21, 2026

Protocol Integer ID: 322255

Funders Acknowledgements:
National Cancer Institute
Grant ID: R01CA259571
National Cancer Institute
Grant ID: U54CA274499

Abstract

Low take rates and inter-tumor variability in growth rates can limit the effectiveness of mouse xenograft models when comparing between groups. To address this problem we developed a simple method to compare multiple cell types within a single mixed xenograft. Individual cell lines or clones were transduced with a lentiviral vector that includes a unique PCR tag, allowing the use of qPCR to determine the proportion of each tagged cell type within a mixed xenograft tumor. We generated vectors with six distinct PCR tags, and two different selectable markers, and have optimized the approach for determining their relative proportions within a mix. An initial pre-amplification step is used to increase the amount of material for subsequent qPCR reactions. This also removes the bulk of the genomic DNA, increasing the specificity of the qPCR step. Samples are then used for qPCR with specific pairs of primers that distinguish between each of the individual PCR tags, and the relative proportion of each tag is determined relative to that in the starting mix. Thus, each tumor provides a direct comparison between individually tagged cell lines or clones. This low
throughput “bar-coding” approach is simple and cost effective and has the potential to reduce the number of animals needed for xenograft experiments.

Guidelines

Tissue collection for this protocol (primary tumors or metastases) needs prior approval by the users' Institutional Animal Care and Use Committee (IACUC) or equivalent ethics committee.

Materials

- HotSHOT lysis solution (25mM NaOH, 0.2mM EDTA, pH 12)
- HotSHOT neutralization solution (40mM Tris-HCl, pH 5)
- Polytron homogenizer
- Pre-amp primers (200nM final of each primer)
- 2x SensiMix SYBR + FITC (Bioline)
- Mango Taq DNA polymerase (Bioline)
- Qiaquick purification kit (Qiagen)
- Bioline Mango Buffer
- MgCl2 (50mM)
- dNTP mix (10mM each dNTP)
- Forward primer (100µM)
- Reverse primer (100µM)
- Bioline Mango Taq
- Qiagen buffer PB
- Qiagen buffer PE
- Qiagen buffer EB
- myIQ thermocycler (Bio-Rad)
- IQ5 software (Bio-Rad)

F) **Primers and vectors**

qPCR tag primers

- PCR tag 1 F: ACAAGTCGGAGTATGTGGA, R: TCTTTTGCTTTGAACTGTCG
- PCR tag 2 F: TACCAGTACAACGCCTCCT, R: TGGGAGTACGGATGCACTTT
- PCR tag 3 F: TGAATGGACACTCTGGGTTG, R: CGATCAGTGAGGTCTTTGG
- PCR tag 4 F: TGAACCAGGAGATCCGAGAC, R: GAAACAGGGCTTTCATTCTGC
- PCR tag 5 F: CCAGATGGAGCCGATATGA, R: TCGTTCTGCTGCTCAATCAC
- PCR tag 6 F: TCCCTCTGTGATGAGCTGTG, R: TCCCTTCTCCTGCGTGCTTC

Pre-amp primers:

- pLenti-puro F: TGATGATGATAAGGCTAGCGG, R: CCGAGGAGAGGGTAGGGAT
- pLX304 F: GGGACAGCAGAGATCCACTTT, R: GGGCCACAACCTCCTATAAA

Internal primers:

- Venus F: CAACTACAACAGCCACAACG, R: GGATCTTGAAGTTGGCCTTG

PCR tag vectors:

- pLenti-puro (Addgene 39481; [2]) was modified by PCR to insert a triple Flag tag, followed by the following restriction enzyme sites: NheI, AscI, PacI and BamHI.
- PCR tags were generated from eYFP by PCR, with six specific pairs of primers and added PacI and BamHI sites, and were inserted into the PacI and BamHI of the modified pLenti-puro.
- A PacI-XhoI fragment from each of the six pLenti-puro tag vectors was inserted into a modified pLX304 vector (Addgene 25890; [3]), removing the CMV promoter and any polylinker cloning sites.
- Sequences were verified by Sanger sequencing and are provided as supplementary information.
- pLX304 was a gift from David Root (Addgene plasmid # 25890; http://n2t.net/addgene: 25890; RRID:Addgene 25890).

- pLenti-puro was a gift from Ie-Ming Shih (Addgene plasmid # 39481; http://n2t.net/addgene: 39481; RRID:Addgene 39481).

Protocol materials

- Mango Taq DNA polymerase, **Bioline**
- Qiaquick purification kit, **Qiagen**
- HotSHOT lysis solution
- HotSHOT neutralization solution

A) HotShot DNA preps

1h 20m

1 Cultured cells:

1.1 Cell pellets collected by scraping or trypsinization were resuspended in PBS at 10^7 cells per ml.

1.2 Pellets can be stored at -20 °C prior to resuspending.

1.3 Genomic DNA was prepared by HotSHOT [1], diluting resuspended cells 1:6 (20 µL + 100 µL) in HotSHOT lysis solution (25 millimolar (mM) NaOH, 0.2 millimolar (mM) EDTA, pH 12), boiling (00:40:00) and neutralizing with equal volume HotSHOT neutralization solution (40mM Tris-HCl, pH 5). 40m

2 Primary tumors:

2.1 For larger samples, such as primary tumors, they were collected, weighed, frozen on dry ice and stored at -20 °C .

2.2 Cecal tumors were homogenized using a Polytron homogenizer at 300 mg/mL in PBS On ice .

2.3 Genomic DNA was prepared by HotSHOT, diluting homogenized tumor 1:6 in HotSHOT lysis solution, as for cell pellets.

2.4 We have also used a similar approach for subcutaneous xenografts. If the primary tumors are very small they could be processed as for small metastases (below).

3 Metastases:

3.1 Smaller samples such as individual metastases were resuspended in ~8 volumes of HotSHOT lysis, boiled for 00:40:00 and neutralized with equal volume HotSHOT neutralization solution. 40m

3.2 Larger metastases can be weighed and treated as for the primary tumors.

B) Optional normalization

4 This optional normalization is not absolutely necessary, but may be useful with larger tumors where differing amounts of the sample may be non-tumor tissue. For cell pellets where approximately equal numbers of cells are present it can be omitted. Additionally, for smaller samples such as individual
metastases we omit this step, and use the HotSHOT sample directly for pre-amplification, without normalization.

5 <u>Pre-amp qPCR normalization:</u>

- 1 µL HotSHOT sample
- 1 µL [M] 5 micromolar (µM) each forward + reverse Pre-amp primers ([M] 200 nanomolar (nM) final of each primer)
- 12.5 µL 2x SensiMix SYBR + FITC (Bioline)
- Water to 25 µL total volume

6 <u>qPCR program:</u>

- 8 minutes, 30 seconds, 95°C
- 40 cycles: 10 seconds, 95°C
  10 seconds, 60°C
  10 seconds, 72°C

7

- Samples were then normalized around an arbitrary Ct of 30 (this can be adjusted if all or most samples cluster well below or above this).
- A Ct of 30 (in this example) would then require 1 µL of HotSHOT for the subsequent pre-amplification reaction, with other samples scaled accordingly.
- The Ct of 30 is designed such that the pre-amplification reaction will bring this into the low to mid 20s range for the Ct for a tag that constitutes half the sample: 12 cycles of pre-amplification, and using only 1/50th of the eluted sample for each qPCR, with each tag starting at 50% in an equal 1 + 1 mix.
- It should also be noted that this normalization does not need to be very accurate, since each sample is internally controlled and a relative proportion determined.
- This just ensures that most qPCR reactions generate a Ct that is in the optimal range for observing differences.

C) Pre-amplification

5m

8

- The PCR tag region is enriched by 12 cycles of pre-amplification PCR with primers common to all six PCR tagging vectors using

Mango Taq DNA polymerase, **Bioline** .

- Pre-amplified PCR product is purified using Qiaquick purification kit, **Qiagen** , and eluted in 50 µL . This eluate can be used directly for qPCR, without further purification or first determining the concentration.

9 Pre-Amplification PCR:

- 1 µL HotSHOT sample
- 4 µL 5x Bioline Mango Buffer
- 0.6 µL $MgCl_2$ ([M] 50 millimolar (mM))
- 0.4 µL dNTP mix (containing 10mM each dNTP)
- 0.2 µL forward primer ([M] 100 micromolar (µM))
- 0.2 µL reverse primer ([M] 100 micromolar (µM))
- 0.2 µL Bioline Mango Taq
- Water to 20 µL total volume

10 PCR program:

- 3 minutes, 94°C
- 12 cycles: 30 seconds, 94°C
- 30 seconds, 60°C
- 30 seconds, 72°C
- 3 minutes, 72°C

11 Pre-amp purification: 5m

- Add 5 volumes Qiagen buffer PB to pre-amp PCR product
- Apply to Qiaquick column
- Wash with 750 µL Qiagen buffer PE
- Add 50 µL Qiagen buffer EB, warmed to 60 °C , incubate 00:05:00
- Spin to elute purified, pre-amplified DNA

D) Quantitative PCR

12 1/50 of the total pre-amplified PCR product per well is used as input for qPCR using SensiMix SYBR + FITC PCR Mix (Bioline) and myIQ thermocycler (Bio-Rad). In general at least duplicate wells are preferred, with separate reactions for each tag in the mixed sample. Ct values are determined by IQ5 software (Bio-Rad).

13 Individual tag qPCR:

- 1 µL HotSHOT sample
- 1 µL 5mM each forward + reverse Tag primers (200nM final each primer)

- 12.5 µL 2x SensiMix SYBR + FITC (Bioline)
- Water to 25 µL total volume

14 qPCR program:

- 8 minutes, 30 seconds, 95°C
- 40 cycles: 10 seconds, 95°C
  10 seconds, 60°C
  10 seconds, 72°C

E) Calculation of relative proportions

15 The proportions of each PCR tag present in the final cell pellets or tumors are calculated by $2^{\Delta Ct}$ using the sample zero (S=0) mixture of cells as the reference. For example, for two tags and a single sample compared to a starting mix:

16 Tag 1: $Ct_{[S1(T1)]}-Ct_{[S0(T1)]}$

$2^{[\Delta Ct(T1)]}$

$2^{[\Delta Ct(T1)]}/(2^{[\Delta Ct(T1)]} + 2^{[\Delta Ct(T2)]})$

= proportion Tag 1 in sample 1

Tag 2: $Ct_{[S1(T2)]}-Ct_{[S0(T2)]}$

$2^{[\Delta Ct(T2)]}$

$2^{[\Delta Ct(T2)]}/(2^{[\Delta Ct(T1)]} + 2^{[\Delta Ct(T2)]})$

= proportion Tag 2 in sample 1

(S0 = time zero starting mix, S1 = sample 1, T1 = Tag 1, T2 = Tag 2)

Protocol references

1. Truett GE, Heeger P, Mynatt RL, Truett AA, Walker JA, Warman ML. Preparation of PCR-quality mouse genomic DNA with hot sodium hydroxide and tris (HotSHOT). Biotechniques. 2000;29: 52, 54.

2. Guan B, Wang TL, Shih Ie M. ARID1A, a factor that promotes formation of SWI/SNF-mediated chromatin remodeling, is a tumor suppressor in gynecologic cancers. Cancer Res. 2011;71: 6718–27. doi:10.1158/0008-5472.CAN-11-1562

3. Yang X, Boehm JS, Yang X, Salehi-Ashtiani K, Hao T, Shen Y, et al. A public genome-scale lentiviral expression library of human ORFs. Nat Methods. 2011;8: 659–661. doi:10.1038/nmeth.1638
